# Mast Cells and Sensory Nerves Contribute to Neurogenic Inflammation and Pruritus in Chronic Skin Inflammation

**DOI:** 10.3389/fncel.2019.00422

**Published:** 2019-09-18

**Authors:** Hanna Siiskonen, Ilkka Harvima

**Affiliations:** Department of Dermatology, Kuopio University Hospital and University of Eastern Finland, Kuopio, Finland

**Keywords:** mast cell, sensory neuron, itch, skin, pruritus

## Abstract

The intimate interaction between mast cells and sensory nerves can be illustrated by the wheal and surrounding flare in an urticarial reaction in human skin. This reaction is typically associated with an intense itch at the reaction site. Upon activation, cutaneous mast cells release powerful mediators, such as histamine, tryptase, cytokines, and growth factors that can directly stimulate corresponding receptors on itch-mediating sensory nerves. These include, e.g., H1- and H4-receptors, protease-activated receptor-2, IL-31 receptor, and the high-affinity receptor of nerve growth factor (TrkA). On the other hand, sensory nerves can release neuropeptides, including substance P and vasoactive intestinal peptide, that are able to stimulate mast cells to release mediators leading to potentiation of the reciprocal interaction, inflammation, and itch. Even though mast cells are well recognized for their role in allergic skin whealing and urticaria, increasing evidence supports the reciprocal function between mast cells and sensory nerves in neurogenic inflammation in chronic skin diseases, such as psoriasis and atopic dermatitis, which are often characterized by distressing itch, and exacerbated by psychological stress. Increased morphological contacts between mast cells and sensory nerves in the lesional skin in psoriasis and atopic dermatitis as well as experimental models in mice and rats support the essential role for mast cell-sensory nerve communication in consequent pruritus. Therefore, we summarize here the present literature pointing to a close association between mast cells and sensory nerves in pruritic skin diseases as well as review the essential supporting findings on pruritic models in mice and rats.

## Introduction

Itching is a very unpleasant sensation that may provoke scratching or the desire to scratch (Savin, 1998). Chronic itch has clinically been classified into 3 groups according to skin changes: (1) pruritus on primary diseased, inflamed skin; (2) pruritus on primary non-diseased, non-inflamed skin; and (3) pruritus with chronic secondary scratch lesions. Pruritus has also been categorized based on underlying diseases as following: (1) dermatological diseases; (2) systemic diseases including diseases of pregnancy and drug-induced pruritus; (3) neurological diseases; (4) psychiatric/psychosomatic diseases; (5) mixed; and (6) others (Ständer et al., 2007b). Based on its duration, itch can be divided into acute (less than 6 weeks) and chronic (more than 6 weeks) (Azimi et al., 2016).

In the skin, the perception of itch originates from the combined action of nervous, cutaneous and inflammatory cells and substances released by them in the microenvironment. In humans, the nerves responsible for the perception of itch also react to stimuli of pain. There are two types of pruriceptors, specialized nerve fibers sensing itch: fastly conducting A-fibers and non-myelinated, slowly conducting C-fibers, which can be further subdivided based on their responsiveness to different chemical or physical stimuli [reviewed in detail in (LaMotte et al., 2014)].

The itch-specific peripheral sensory neurons may be a subgroup of nociceptive neurons. Subsets of mechano-insensitive nociceptive C-fibers can respond to histamine resulting in the release neuropeptides, including substance P and calcitonin gene -related peptide (CGRP). Instead, the mechano/heat-sensitive nociceptors are not reactive to histamine, but show a response to a cowhage plant protease, leading to stinging itch, which is possibly mediated via protease-activated receptor-2 or -4 (PAR-2 or -4). This type of stinging itch resembles that in skin inflammatory diseases. Nevertheless, cutaneous nerves contain a range of different receptors and ion channels. Therefore, different combinations of receptors may produce a certain type of itch and/or pain sensation. In the spinal dorsal horn, gastrin-releasing peptide-expressing neurons participate in the transmission of itch (Albisetti et al., 2019). The sensing system is further complexed by excitatory interneurons, spinothalamic tract neurons and the central nervous system where these sensations are recognized (Baraniuk, 2012; LaMotte et al., 2014; Talagas et al., 2018). Furthermore, it is noteworthy that chronic inflammation in skin causes additional changes to the neural and proinflammatory cell network.

Free nerve endings in the epidermis are important for sensing itch. It has been proposed that there is a reciprocal synaptic-like interaction between nerves and keratinocytes (Talagas et al., 2018). One possible mediator in this interaction is ß-endorphin, as keratinocytes expressing this mediator have been found around unmyelinated fibers that can be activated via μ-opioid receptor (Bigliardi-Qi et al., 2004). In fact, the whole complex of nerve fibers and the epidermal cells have been proposed to form the “itch receptor” (Greaves, 2010). The skin cells themselves express a wide array of mediators and their receptors involved in the perception of itch (Greaves, 2010). In addition to nerve fibers and epidermal cells, the cells of the immune system constitute yet another part in pruritus. In regard to cellular events in itching skin, mast cells are important players that are involved in neurogenic inflammation and its associated itch. In this review, the fundamental role of mast cells and sensory nerves in itch is discussed.

## ITCH-Related Properties of Mast Cells

Mast cells are important effector cells in allergic reactions and immunity [reviewed in Conti et al. (2017)], but they also contribute to carcinogenesis (Biswas et al., 2014; Hu et al., 2018; Saadalla et al., 2018). Mast cells are abundant in body-environment interfaces in the skin and the gastrointestinal tract (Gurish and Austen, 2001) as well as present in the meninges of the central nervous system (Bo et al., 1992; Theoharides et al., 2005), and in the lung (Bradding et al., 2006). In the skin, mast cell numbers are highest in the upper dermis and their numbers are not affected by age or sex (Weber et al., 2003), but increase in response to various environmental stimuli as shown after ultraviolet radiation in human skin (Grimbaldeston et al., 2003; Kim et al., 2009), and after exposure to topical sensitizing agents in mice (Kitagaki et al., 1995; Tomimori et al., 2002). It is thus obvious that mast cells constitute an inherent component of itch.

Mast cells are fully loaded with preformed mediators or they produce newly-synthesized mediators, including proteases, histamine, lipid-derived mediators, cytokines, and chemokines which they release upon activation through a variety of mechanisms leading to degranulation, piecemeal degranulation, and/or mediator secretion without degranulation [reviewed in da Silva et al. (2014) and in Gupta and Harvima (2018)]. In type I hypersensitivity, mast cell degranulation is a fundamental event and involves crosslinking of an antigen with two IgE molecules bound to FcεRI receptors (Owen et al., 2013). Mast cell activation has been studied especially in the context of urticaria. It is known that several factors such as infections, stress, certain foods, pseudoallergens, hormones, neuropeptides, and Th2 inflammation may prime mast cells [reviewed in Church et al. (2018)]. Also autoimmune mechanisms may lead to mast cell activation. In these cases, IgE recognizes dermis-derived autoantigens, of which more than 200 are present in chronic spontaneous urticaria (Schmetzer et al., 2018). Another mechanism is the presence of IgG or IgM class antibodies against IgE (Gruber et al., 1988) or against its FcεRI receptor (Hide et al., 1993). These autoantibodies may also target the eosinophils, which release mast-cell activating factors (Puccetti et al., 2005).

## ITCH-Related Factors Released by Mast Cells

Mast cells release the contents of their secretory granules to their surroundings upon degranulation. Many of these granule mediators or mediators synthesized *de novo* (Harvima et al., 2014) participate in the development of itch.

### Histamine and Its H1 and H4 Receptors

Histamine is the most important pruritogenic mediator of mast cells. Histamine has four receptors, namely H1–H4, of which H1 and H4 are important in pruritus. The function of these receptors in itch has been mainly studied in mouse models, and it has been shown that skin sensory neurons express H1, H3 and H4 (Rossbach et al., 2011). In mouse models, H1-antagonists have been effective in decreasing itch, which has been known already for decades (Sugimoto et al., 1998), although H4-antagonists (Dunford et al., 2007; Yamaura et al., 2009) have proved to be more potent. Histamine acts also on Transient receptor potential vanilloid receptor-1 (TRPV-1) in sensory neurons (Shim et al., 2007). In keratinocytes, also TRPV-4 is a histaminergic pruriceptor (Chen et al., 2016).

### Tryptase and PAR-2

Tryptase, one of the main proteinases secreted by mast cells, can induce pruritus in mice and its effects are inhibited by PAR-2 antibody or PAR-2 antagonist, showing that PAR-2 is involved in tryptase-induced pruritus (Ui et al., 2006). Involvement of tryptase and PAR-2 in itch has also been reported in a mouse model of atopic dermatitis (Zhu et al., 2015). In line with these data, non-lesional and lesional skin biopsies from patients with atopic dermatitis show PAR-2 in sensory nerves with closely located mast cells (Steinhoff et al., 2003).

### IL-31 and Its Receptor IL-31RA

Interleukin-31 (IL-31) is important in the pruritus of atopic dermatitis (Sonkoly et al., 2006) and it also participates in the itch of cutaneous lymphoma (Nattkemper et al., 2016). IL-31 has been shown to increase the growth and sprouting of cutaneous sensory nerves (Feld et al., 2016), which express its receptor, IL-31RA (Cevikbas et al., 2014).

Interleukin-31 has been demonstrated to induce mild itch that appears slowly starting at 143 min after skin prick test, which is associated with a long-lasting erythema. By comparison, histamine induces immediate itch that starts within 5 min after skin prick test (Hawro et al., 2014). Human mast cells (Niyonsaba et al., 2010; Petra et al., 2018) and T-cells (Dillon et al., 2004) are sources of IL-31 in skin, thus participating in the development of itch. Moreover, mast cell-derived histamine in addition to IL-31 increase the secretion of brain-derived natriuretic peptide, which in turn affects dendritic cells and keratinocytes to produce cytokines and other mediators, leading to inflammation, and increased itch signaling (Meng et al., 2018).

### Leukotrienes and Prostaglandins

Leukotrienes and prostaglandins are also involved in itch, but by different mechanism. When administered intradermally, leukotriene B4 induces itch while prostaglandin E2 does not (Andoh and Kuraishi, 1998). Leukotriene B4 is released from keratinocytes in response to PAR-2 receptor activation (Zhu et al., 2009) and it is involved in the itch-causing cascades of substance P (Andoh et al., 2001) and IL-31 (Andoh et al., 2017a). PAR-2 activation and leukotriene B4 release participate also in dermatophyte-induced itch (Andoh et al., 2014). In addition to producing leukotriene B4 by themselves (Satpathy et al., 2015), human, and murine mast cells also express leukotriene B4 receptors BLT1 and BLT2 (Lundeen et al., 2006). On the contrary, prostaglandin D2, also produced by mast cells themselves (Murakami et al., 1995), decreases histamine release from mast cells and inhibits scratching in a mouse model (Hashimoto et al., 2005). Thus, it seems that mast cells release many mediators that also control their own function.

### Neuropeptides and Mast Cell Activation

There are several neuropeptides released by the sensory neurons in the skin, which then activate mast cells. Mast cells degranulate in response to nerve growth factor (NGF) and this signaling acts through TrkA tyrosine receptor (Horigome et al., 1993). Interestingly, mast cells can secrete NGF also by themselves suggesting an autocrine or paracrine mechanism (Nilsson et al., 1997).

The potency of substance P in degranulating mast cells and causing itchy wheals was found already decades ago (Hägermark et al., 1978). The effects of substance P on mast cells are mediated either through neurokinin-1 receptor (NK-1R) or Mas-related G protein coupled receptor-X2 (MRGPRX2) (Kulka et al., 2008; Subramanian et al., 2016). In the initial phases of topical therapy with calcineurin inhibitors pimecrolimus and tacrolimus, pruritus and burning is often present. These compounds have been shown to release substance P and CGRP from primary afferent nerve endings in murine skin, leading to mast cell degranulation and thus release of pruritogenic histamine, and tryptase (Ständer et al., 2007a). Mast cells degranulate rapidly in response to substance P resulting in wheal reaction in the skin (Huttunen et al., 1996; Yamaoka and Kawana, 2007). The ability of substance P to induce histamine release seems to appear only at high concentrations (Weidner et al., 2000). In line with this, substance P decreases mast cell recruitment and degranulation when used as a topical treatment in a murine model of atopic dermatitis (Choi et al., 2018), pointing to a concentration-dependent dual role of substance P.

Interestingly, stimulation of human mast cells with substance P and an IL-1 family member, IL-33, increase the secretion of proinflammatory TNF and IL-1β and these responses are inhibited by a natural flavonoid, methoxyluteolin (Taracanova et al., 2017, 2018). A pure luteolin with Ashwagandha has been proposed as a relief to patients suffering from stress and inflammation-associated diseases (Theoharides and Kavalioti, 2018). This release of cytokines is potentially inhibited also by IL-37, another IL-1 family cytokine [reviewed in Caraffa et al. (2018), Tettamanti et al. (2018), Gallenga et al. (2019)]. The effect of substance P to release proinflammatory cytokines from mast cells points to a mechanism how mast cells participate in the neurogenic inflammation in psoriasis and atopic dermatitis as discussed below in more detail. Recently, Nakamura et al. (2019) reported increased levels of IL-33 in the stratum corneum of patients with atopic dermatitis. All these data emphasize the role of these cytokines in the context of neuroinflammation and itch, in which mast cells obviously participate.

Vasoactive intestinal peptide (VIP) is another potent neuropeptide to degranulate mast cells (Fjellner and Hägermark, 1981; Huttunen et al., 1996), a reaction mediated through VPAC2, and/or MRGPRX2 receptors on mast cells (Kulka et al., 2008; Subramanian et al., 2016). In addition, substance P and VIP have been found to stimulate human mast cells *in vitro* conditions to release cytokines and chemokines, including TNF-α, GM-CSF, IL-3, CCL2, CCL5, CXCL8, CXCL9, and CXCL10 (Kulka et al., 2008).

## Mast Cells, Skin Diseases, and ITCH

In a retrospective study conducted by Sommer et al. (2007), dermatological disease was the probable cause of the itch in 41,8 % of patients, while almost a similar number of cases (44,8 %) showed no apparent origin of the symptoms. The role of mast cells in the development of itch has been studied mostly in psoriasis, atopic dermatitis and urticaria. It is likely that the increased contacts between nerve fibers and mast cells often seen in these dermatoses constitute the morphological basis for itch chronicization during chronic skin inflammation. The current understanding of the role of mast cells and sensory nerves in itch in these and selected other pruritic dermatoses is discussed here next.

### Mast Cells, Sensory Nerves, and Itch in Psoriasis

Psoriasis, a common chronic inflammatory and scaly skin disease, is characterized by pruritus that affects 60–90% of the patients and can appear in different forms, such as stinging, pinching, tickling, crawling, burning or pain sensations (Szepietowski and Reich, 2016). On the other hand, psychosocial stress can exacerbate psoriasis in 40–80% of the patients (Basavaraj et al., 2011). Thus, pruritus and stress may be reciprocally interconnected factors in psoriasis.

Emotional stress is associated with the activation of a variety of neuro-immune-endocrine systems. For example, the hypothalamic-pituitary-adrenal (HPA) axis is activated and stress hormones are released, including corticotroping-releasing hormone (CRH), adrenocorticotropic hormone, and glucocorticoids. Interestingly, human skin has its own functional peripheral equivalent of the HPA axis. Furthermore, numerous other factors are activated in stress, such as α-MSH, neuropeptides, neurotrophins and the sympathetic nervous system (Arck et al., 2006).

Several studies have previously shown that sensory nerve fibers and neuropeptides, including substance P, neurokinin A and VIP, are increased in the psoriatic lesion (Naukkarinen et al., 1989; Eedy et al., 1991; Amatya et al., 2011). Furthermore, the morphologic contacts between neurofilament^+^ nerves and tryptase^+^ mast cells are more frequent in psoriatic lesions than in non-lesional psoriatic skin or normal skin (Naukkarinen et al., 1991, 1993). Even the morphologic contacts between substance P^+^ and CGRP^+^ fibers and tryptase^+^ mast cells, but not the contacts between VIP^+^ fibers and tryptase^+^ mast cells, have been found to be increased in the psoriatic lesion. Therefore, there is a morphologic basis for mast cell-neural interaction as well as neurogenic inflammation in psoriasis.

The increase in mast cell numbers, especially the MC_TC_-type (tryptase^+^ and chymase^+^) of mast cell, in the psoriatic lesion has been known for decades. However, in contrast to the resistant tryptase, chymase is sensitive to the action of serum protease inhibitors, which may explain the finding that the enzyme activity of chymase is decreased in the psoriatic lesion (Harvima et al., 2008). The net outcome of the partial inactivation of chymase may be an uncontrolled and enhanced substance P-mediated neurogenic inflammation, as chymase degrades substance P and VIP, but tryptase degrades VIP and CGRP (Caughey et al., 1988; Franconi et al., 1989; Walls et al., 1992). Tryptase has the capability to cleave and activate the PAR-2 receptor. Therefore, the serine proteinase may not only activate the receptor on nerves and numerous proinflammatory cells but it may activate PAR-2 on mast cells themselves in a para- or autocrine fashion, as the percentage of tryptase^+^ mast cells containing PAR-2 immunoreactivity is increased in the psoriatic lesion (Carvalho et al., 2010). Furthermore, the activation of PAR-2 sensitizes TRPV-1 leading to increased substance P and CGRP release (Amadesi et al., 2004) and TRPV-1 is expressed in substance P^+^ fibers as well as in mast cells in human skin (Ständer et al., 2004).

In addition to neuropeptides, the emotional stress in psoriasis may transmit its signals to the skin through CRH and CRH-R1 receptor on mast cells as suggested previously (Harvima and Nilsson, 2012). This hypothesis is supported by a report that the immunostaining of CRH is increased in the epidermis, sweat glands, and hair follicles in the psoriatic lesion (Kim et al., 2007). In addition, we have analyzed the expression of CRH-R1 immunoreactivity in mast cells in psoriasis and found that the percentage of tryptase^+^ mast cells expressing CRH-R1 is higher in the lesional than non-lesional skin of 8 psoriatic patients (Haimakainen S et al., unpublished results).

There are several studies that have investigated molecular differences in skin between pruritic and non-pruritic-type of psoriasis. For example, the pruritic-type of psoriasis is characterized by increased levels of substance P and nerve fibers; decreased levels substance P-degrading neutral endopeptidase; increased levels of NGF and/or its receptor TrkA (Nakamura et al., 2003; Chang et al., 2007; Amatya et al., 2011), decreased expression of semaphorin-3A (an axon-guidance molecule) (Taneda et al., 2011; Kou et al., 2012), and increased numbers of total and degranulated mast cells (Nakamura et al., 2003).

### Mast Cells, Sensory Nerves, and Itch in Atopic Dermatitis

Atopic dermatitis (AD) is a well-known chronic eczematous skin disease characterized by distressing pruritus that can be exacerbated by inflammatory mediators, sensory nerves, skin dryness, heat, sweat, and emotional stress (Suarez et al., 2012; Murota and Katayama, 2017).

Like in the case of psoriasis, mast cells and sensory nerves have been suggested to play a role in neurogenic inflammation and itch in AD. Regarding mast cells, there are similarities between these diseases. In the lesional atopic dermatitis skin, the number of tryptase^+^ mast cells is increased slightly, but the activity of chymase is decreased (Järvikallio et al., 1997; Ilves and Harvima, 2015). Like in psoriasis, the possible explanation for the partial inactivation of chymase in AD is the presence of chymase inhibitors in mast cells (Saarinen et al., 2001). The reduced chymase activity means that the enzyme cannot degrade and control efficiently a variety of proinflammatory peptides and proteins, including IL-6, IL-13, TNF-α, IL-4, IL-5, substance P, and VIP (Franconi et al., 1989; Tunon de Lara et al., 1994; Zhao et al., 2005).

This is relevant for the inflammation, as the percentage of mast cells expressing TNF-α, IL-4, IL-6, and CD30 ligand is increased in the lesional AD skin (Horsmanheimo et al., 1994; Ackermann and Harvima, 1998; Fischer et al., 2006; Ilves and Harvima, 2015). In addition, the severity of itching and tryptase^+^ and IL-6^+^ mast cells correlate inversely with the (pro)filaggrin immunostaining in the epidermis of AD skin (Ilves et al., 2015).

Like in the case of psoriasis, there is an increased sensory nerve density in the epidermis and dermis in AD lesions, which suggests an enhanced neurogenic inflammation (Tobin et al., 1992; Urashima and Mihara, 1998; Järvikallio et al., 2003; Tominaga et al., 2009; Kubanov et al., 2015). Previously, it has been demonstrated that there is an increased expression of NGF and amphiregulin in the epidermis, but decreased expression of nerve repulsion factors, semaphorin-3A and anosmin-1, in AD lesions, which can explain the increased nerve density in AD (Tominaga et al., 2009; Tominaga and Takamori, 2014; Kubanov et al., 2015). In addition, another explanation for the increased nerve density is the finding that the expression of NGF is elevated in mast cells in AD lesions, and mast cells can express the receptor of NGF, i.e., TrkA (Nilsson et al., 1997; Xiang and Nilsson, 2000; Groneberg et al., 2005). There are also other regulatory mechanisms between NGF and tryptase, as NGF treatment of mast cells *in vitro* can increase the expression of TrkA as well as the contents of tryptase and histamine (Groneberg et al., 2005), and tryptase can generate mature NGF from pro-NGF (Spinnler et al., 2011). These molecular interactions can explain, at least in part, the finding that the morphologic contacts between tryptase^+^ mast cells and neurofilament^+^ fibers are increased in AD lesions (Järvikallio et al., 2003).

Acute psychologic stress can result in marked changes in nerves, neuropeptides and mast cells. For example, the exposure of AD patients to acute Trier social stress test (TSST) associated with decreased NGF^+^ and PGP9.5^+^ fibers and decreased contacts between PGP9.5^+^ fibers and tryptase^+^ mast cells in AD lesions. However, in the non-lesional AD skin, these parameters increased, rather than decreased. Moreover, a positive correlation was observed between itch and mast cell-nerve fiber contacts in the non-lesional AD skin after TSST or lesional AD skin before TSST (Peters et al., 2014).

The classic histaminergic itch is mediated via H1-receptors on cutaneous sensory nerves, though H4-receptors on nerves can also be involved in pruritus (Ohsawa and Hirasawa, 2014). However, antihistamines usually are not effective in relieving distressing pruritus in chronic skin inflammation. The reason is the non-histaminergic itch that can be mediated through other distinct pathways. The PAR-2 activation on sensory fibers is one of them, as PAR-2^+^ fibers are increased in AD lesions. Therefore, tryptase released from mast cells can activate PAR-2 on nerve fibers with subsequent release of substance P and CGRP and potentiation of neurogenic inflammation (Steinhoff et al., 2003; Kempkes et al., 2014). Interestingly, lichenified lesions of atopic dermatitis showed no staining for substance P or VIP nor degranulated mast cells, suggesting that other factors may contribute to pruritus in chronic, lichenified lesions (Urashima and Mihara, 1998).

Interleukin-31 can represent another pathway for non-histaminergic itch in AD. This cytokine is derived from Th2 cells and mast cells. In the psoriatic and AD lesions, mast cells show increased levels of IL-31 immunoreactivity compared to normal skin (Niyonsaba et al., 2010). The expressions of the mRNA of IL-31 and its receptor IL-31RA are increased in AD, but not psoriatic, lesions. Furthermore, the receptor is located in small-diameter neurons of human dorsal root ganglia (Sonkoly et al., 2006; Cevikbas et al., 2014).

Interleukin-31 may not only induce slowly developing itch (Hawro et al., 2014), but it may also increase the elongation, branching and density of nerves in AD lesions (Feld et al., 2016).

### Mast Cells, Sensory Nerves, and Itch in Urticaria

Urticaria is a fundamentally mast cell-driven disease, in which pruritus is a key symptom in addition to wheals and/or angioedema (Zuberbier et al., 2018). In urticaria, it is the degranulation of mast cells and release of their mediators that cause the symptoms by activating sensory nerves and causing vasodilatation and plasma extravasation (Zuberbier et al., 2018). Although the details of mast cell activation in urticaria are not known yet, mast cells may be activated in urticaria by allergens/autoallergens or IgG antibodies against IgE or its receptor. In patients with chronic urticaria, more than 200 autoantigens reacting with IgE have been detected and of these, IL-24 is a common and functional autoantigen recognized by IgE antibodies (Schmetzer et al., 2018).

Interestingly, also a plethora of other agents are known to prime mast cells for further activation [reviewed in Church et al. (2018)]. Recently, a mouse model showed that Mrgprb2, the ortholog of the human MRGPRX2, and might be a mast cell-specific key receptor in pseudoallergic reactions (McNeil et al., 2015). This receptor is upregulated in the skin of patients with severe chronic urticaria (Fujisawa et al., 2014).

### Mast Cells, Sensory Nerves, and Itch in Selected Other Dermatoses

Prurigo nodularis (PN) is a typical example of an itchy dermatosis. The close contact of mast cells and nerve fibers in PN lesions was reported already 20 years ago by Liang et al. (1998). They also noticed that there are increased mast cell numbers in the lesions. There are several other pruritic skin diseases, in which the changes in mast cell numbers have been investigated and they definitely participate in the pathogenesis, although the exact details of their role in pruritus in these dermatoses is not clear yet. Mast cell numbers are increased already in the perilesional skin in patients with hidradenitis suppurativa (HS) and the numbers are further elevated in early and chronic lesions (van der Zee et al., 2012). In line with this finding, about 60 % of patients with HS report moderate or severe pruritus (Matusiak et al., 2018). Similarly, more than one third of patients with basal cell carcinoma or squamous cell carcinoma report to have pruritus (Mills et al., 2012; Yosipovitch et al., 2014) and elevated mast cell numbers in both tumors have been reported (Diaconu et al., 2007; Haimakainen et al., 2017). The connection between mast cell numbers and itch is not so clear in melanoma, in which less patients report itch (Yosipovitch et al., 2014) and both increased (Toth-Jakatics et al., 2000; Ribatti et al., 2003a, b), and decreased (Biswas et al., 2014; Siiskonen et al., 2015; Rajabi et al., 2017) mast cell numbers have been reported. However, since the exact functions of mast cells in skin cancers are not known at the moment, also their role in itch remains unclear.

### Animal Models in Research on Mast Cells and Itch

The growing research performed in experimental animals has revealed that the molecular mechanisms of pruritus as well as the ligands and receptors involved in itch induction are more complex than thought, and it is beyond the scope if this review to describe them all.

Animal models to study pruritus have been mainly developed for AD. Subcutaneous capsaicin injections to neonatal rats causes long-lasting, pruritic skin changes with histopathological findings resembling AD, including increased mast cell number (Back et al., 2012). The Kyoto Fancy Rat Stock 4 is another rat model of AD showing spontaneous itchy dermatitis with increased transepidermal water loss and mast cell numbers (Kuramoto et al., 2015). The bile duct ligation rat model of liver disease has provided a good insight to neuroinflammation and itch. In these rats, PAR-2 receptors are activated and this potentiates the TRPV-1 channels (Belghiti et al., 2013). As discussed above, mast cell tryptase may activate PAR-2, pointing to a role for mast cells also in hepatic pruritus. Elevated tryptase and PAR-2 levels are also found in a mouse model with ovalbumin-induced AD-like dermatitis (Zhu et al., 2015). Mast cells and sensory nerves can be found in apparent morphologic contact in the skin. For example, in a mouse model of hapten-induced AD, mast cells in the lesional skin express high levels of cell adhesion molecule-1 (CADM-1), a molecule that was found to enhance adhesion and communication between sensory nerves and mast cells *in vitro* (Hagiyama et al., 2013). Human mast cells have also been found to express CADM-1 that probably interacts with nectin-3 on nerves (Furuno et al., 2012; Moiseeva et al., 2013).

The interaction between nerve fibers and mast cells can be affected by molecules leading to decreased itch and inflammation. In the NC/Nga mouse model of AD, dorsal skin lesions develop spontaneously. The treatment of these lesions with intracutaneous injections of semaphorin-3A, a nerve repulsion factor, for 5 days followed by biopsing on day 11 (Yamaguchi et al., 2008) revealed a significant reduction in the clinical skin score and scratching behavior, decrease in PGP9.5^+^ nerve fibers in the epidermis, decrease in mast cell numbers, infiltrating CD4^+^ T cells, IL-4 production, and epidermal thickness (Yamaguchi et al., 2008). In addition, oral administration of chymase inhibitor has been shown to ameliorate dermatitis (Watanabe et al., 2007) as well as scratching behavior in these NC/Nga mice (Terakawa et al., 2008).

In pruritus, the neuroendocrine signals may travel from the central nervous system to periphery and vice versa. MacQueen et al. (1989) showed in 80’s that rats exposed to both audiovisual cue and antigen injection were conditioned to mast cell protease II release after reexposure to the audiovisual cue only. A sonic stress for 24 h in mice can induce degranulation of mast cells, changes in endothelium, increase in substance P^+^ fibers and their contacts with mast cells, and increase in the expression of NGF in skin mast cells (Peters et al., 2005, 2011; Pavlovic et al., 2008). Furthermore, skin mast cells in rats can be activated by stress induced by immobilization (Singh et al., 1999). In summary, there is compelling *in vivo*-evidence that show activation of mast cells induced by stress.

In an AD model induced by IL-13 in mice, the scratching behavior evoked by itch associated with increase in PGP9.5^+^, CGRP^+^, and TRPA-1^+^ (transient receptor potential ankyrin (1) nerves, mast cells, and particularly in TRPA-1^+^ mast cells. However, there was no increase in TRPV-1 in inflamed skin. In line with this, TRPA-1^+^ mast cells and nerves and the contacts between them were increased in the lesional skin of patients with AD (Oh et al., 2013). Therefore, this study emphasizes the role of TRPA-1 in itch associated with skin inflammation. In contrast to this, itch evoked by intradermal injections of β2-microglobulin in mouse skin is related, at least in part, to TRPV-1^+^ primary sensory nerves (Andoh et al., 2017b). Furthermore, itch evoked by IL-31 injections in mice is related to both TRPV-1 and TRPA-1 using knockout mouse models (Cevikbas et al., 2014). In addition, itch induced in mice by lysophosphatidic acid-injections is related to both cation channels (Kittaka et al., 2017).

## Conclusion and Future Challenges

Despite thorough and extensive research on mast cells during the past decades, these cells still remain to be an intriguing, complex cell type present in our body-environment interfaces. Although mast cells are physiologically meant to stay alert and to react to potentially noxious agents and conditions, their reactions may turn harmful. In several disorders, their functions seem to increase itch, which is not always beneficial to the host.

**FIGURE 1:**
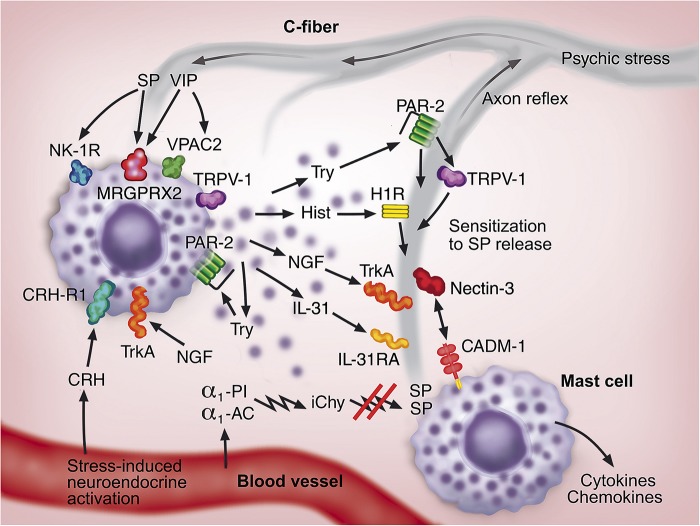
A hypothetical model for the communication between mast cells (two mast cells shown in purple) and sensory nerves (shown in gray) in neurogenic inflammation in the skin. The neuroendocrine and neural systems are activated as a consequence of psychosocial stress. The signals traveling through C-fibers lead to the release of neuropeptides, substance P (SP) and vasoactive intestinal peptide (VIP), from C-fiber endings. The increased cutaneous blood flow conveys corticotropin-releasing hormone (CRH) to the developing inflammation. These neuroendocrine factors activate mast cells through the receptors NK-1R, VPAC2, MRGPRX2, and CRH-R1. Tryptase (Try), histamine (Hist), NGF, IL-31 released from mast cells activate their corresponding receptors PAR-2, H1R, TrkA, and IL-31RA, respectively, on C-fibers. Furthermore, mast cells themselves are activated through PAR-2 and TrkA in an auto- or paracrine fashion. Mast cell-derived mediators activate C-fibers leading to the spread of signal, which can also take place through an axon reflex-related mechanism. NGF and IL-31 support the growth of C-fibers. Chymase released from mast cells is susceptible to the inactivation by serum protease inhibitors, α_1_-proteinase inhibitor (α_1_-PI) and α_1_-antichymotrypsin (α_1_-AC), resulting in inactivated chymase (iChy) that cannot degrade and inactivate SP. PAR-2 can sensitize the capsaicin receptor (TRPV-1) in C-fibers enhancing SP and CGRP release. The intimate functional and morphologic communications between mast cells and C-fibers are further strengthened by the cell adhesion molecule-1 (CADM-1) on mast cells and nectin-3 on C-fibers. As a consequence of these multiple interactions, a feedforward loop is developed, which leads to increase in mast cells and C-fibers, development of vicious circle, and potentiation of neurogenic inflammation and itch.

The complex functional and morphologic interaction between mast cells and sensory nerves is summarized and illustrated in Figure 1. Psychic stress can aggravate itch in several skin diseases. Although the mechanism is not clear in detail, the activation of the systemic HPA axis and/or its equivalent in the skin as well as the activation of neural response can play a role in activating the neurogenic inflammation in the skin. As a consequence, several neuroendocrine mediators, including CRH, substance P and NGF, are released to the circulation and/or are produced locally in the skin. These mediators lead to activation of mast cells and release of their proinflammatory mediators that modify the inflammation of the skin disorder, often increasing itch. The mechanisms are complex owing to the intimate reciprocal communication between mast cells and sensory nerves resulting in increase in mast cell numbers and nerve fibers, development of a vicious circle, and exacerbation of neurogenic inflammation and pruritus.

Future research should aim at further elucidating the details of mast cell biology in different physiological and pathological skin conditions in humans. Here, it will be important to focus on the association of psychic stress with mast cell-neural functions in patients with psoriasis or atopic dermatitis under standardized stress conditions. We should also aim at more in-depth understanding of mast cell heterogeneity and recruitment to the skin and how these may affect mast cell-neural responses in chronic pruritic disorders. In addition, the inhibitors of mast cell mediators or drugs preventing mast cell activation (Harvima et al., 2014) may provide new therapeutic options to treat distressing itch and thereby improve the quality of life of patients.

## Author Contributions

Both authors wrote and edited the text.

## Conflict of Interest Statement

The authors declare that the research was conducted in the absence of any commercial or financial relationships that could be construed as a potential conflict of interest.
